# A Novel Technique for the Treatment of Inferior Pole Fractures of the Patella: A Preliminary Report

**DOI:** 10.1111/os.13518

**Published:** 2022-10-04

**Authors:** Zhixiang Gao, Nengji Long, Kai Yao, Peng Cai, Yixin Dai, Wei Yu, Cong Xiao

**Affiliations:** ^1^ Department of Orthopaedics The Third Hospital of Mianyang· Sichuan Mental Health Center Mianyang China

**Keywords:** fracture, inferior pole, Krackow suture, Nice knots, patellar

## Abstract

**Objective:**

Most inferior pole fractures of the patella are comminuted. Therefore, an ideal treatment method has not been determined. We have presented a modified tension band fixation technique—the Krachow suturing, Nice knot combined with tension band fixation—and reported the results of the procedure.

**Methods:**

A total of 16 inferior patellar pole fractures were treated at our institution between January 2019 and October 2020, 15 of which underwent treatment with the modified tension band fixation technique consisting of Krachow suturing with Nice knots combined with tension band fixation. The primary measures: knee motion, Bostman score, anterior knee pain, fixation failure.

**Results:**

Bone union occurred at a mean of 9 weeks postoperatively (range: 8–13). There were no cases of postoperative anterior knee pain, refracture of the inferior patellar pole or wire breakage. The patients regained full ROM of the knee joint without functional deficits during follow‐up; the mean ROM was 128.46° ± 7.07° (range: 113.4°–137.8°). At the last follow‐up, all patients had a mean Bostman score of 28.40 ± 1.29 (range: 26–30), with an excellent score in 11 patients and a good score in four patients.

**Conclusion:**

The modified tension band fixation technique for the treatment of inferior patellar pole fractures is a simple and easy‐to‐perform surgical technique that provides stable fixation and good functional results.

## Graphical abstract

Most inferior patellar pole fractures are comminuted, so it is difficult to achieve anatomical reduction and strong fixation. As a result, many investigators have proposed different surgical techniques and devices to treat inferior patellar pole fractures, such as partial patellectomy, screw fixation with a titanium cable or steel wire, interwoven sutures and basket plates, mesh plates, angle‐stable locking plates, wire interwoven sutures and Krachow sutures, and separate vertical wiring (SVW). The modified tension band fixation technique (Krachow suturing with Nice knots combined with tension band fixation) for the treatment of inferior patellar pole fractures is a simple and easy‐to‐perform surgical technique that provides stable fixation and good functional results.

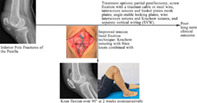

## Introduction

The patella is the largest sesamoid bone in the body and is located at the anterior part of the knee joint. Patellar fractures are common and account for approximately 1% of all fractures^1^. The patellar surface has seven main articular surfaces, which are divided into the superior articular surface and the inferior nonarticular surface, with a vertical ridge further subdividing the superior part into the medial and lateral surfaces^2^. The inferior pole of the patella, with no articular surface coverage, is located in the distal 1/4 of the patella. Fractures of the inferior pole of the patella are rare and account for 9.3%–22.4% of patellar fractures^3^, ^4^.

Inferior pole fractures of the patella are usually combined with tears of the joint capsule and can result in complete damage to the knee extensor mechanism. Most inferior patellar pole fractures are comminuted, so it is difficult to achieve anatomical reduction and strong fixation. Inappropriate treatment can result in patellar height loss and seriously reduce the function of the knee extensor device. As a result, many investigators have proposed different surgical techniques to treat inferior patellar pole fractures, such as partial patellectomy^5^, screw fixation with a titanium cable or steel wire^6^, interwoven sutures and basket plates^7^, ^8^, ^9^, mesh plates^10^, angle‐stable locking plates^11^, ^12^, ^13^, Novel Rim Plating Technique^14^, wire interwoven sutures and Krachow sutures^15^, Anchor and Krackow‐“8” Suture^16^, Novel Tension Band and Patellotibial Tubercle Cerclage^17^, and separate vertical wiring (SVW)^18^. In these techniques, partial patellectomy leads to patella baja, which disrupts the biomechanical anatomical relationships of the normal patellofemoral joint^19^, ^20^. As a consequence, the vertical vector forces into the patellofemoral joint increase, which further accelerates the degeneration of the patellofemoral joint^20^, ^21^. Therefore, in a review of early foreign literature, partial patellectomy was not recommended for inferior patellar pole fractures^1^, ^5^. In contrast to partial patellar resection, fixation methods such as “mesh plates, basket plates” do not require additional bracing to immobilize the knee after surgery, and the second method allows for functional knee exercises and early weight bearing immediately after surgery^7^, ^8^, ^9^, ^10^. Although various special plates (mesh plates, basket plates, angle‐stable locking plates) have been proposed to treat inferior patellar pole fractures, these plates cause complications such as patellar ligament injury and screw failure during knee flexion activities and are not available in many countries. Hence, Yang *et al*.^18^ used the SVW technique to treat 25 patients with inferior pole patellar fractures with a mean postoperative follow‐up of 22 months, and the Bostman score reached 29.5, but this technique is prone to postoperative fixation failure in patients with osteoporosis and comminuted fractures of the inferior pole of the patella. To date, it is still controversial as to the ideal treatment for inferior pole patellar fractures. Is there a simple and easy‐to‐grasp technique to treat inferior pole fractures of the patella? We propose a modified tension band technique.

In 1979, Müller *et al*.^22^ proposed the tension band fixation technique, which consists of two parallel Kirschner wires (K‐wire) and a circular wire to compress the fracture line and cause compression of the articular surface by tension forces and achieved good clinical results in the treatment of patellar fractures. Therefore, tension band fixation is still one of the most commonly used fixation techniques in the treatment of patellar fractures^23^. Tension band fixation can resist a maximum load of 395 N, which is approximately 79 N higher than the load generated by quadriceps extension^24^. However, due to the unique structure of the inferior pole of the patella and the fact that most fractures are comminuted, simple tension band fixation may fail easily.

We applied a new technique, modified tension band fixation (Krachow suturing with Nice knots combined with tension band fixation) for the treatment of inferior pole fractures of the patella. Nice knots and Krachow sutures were used to counteract the tension generated by the quadriceps muscle prior to tension band implantation. The purpose of our study was to review the clinical outcomes of patients treated with this modified tension band fixation technique.

## Materials and Methods

Inclusion criteria were as follows: (i) unilateral inferior pole of patella fracture; (ii) AO/OTA type 34‐A1; (iii) minimum length of follow‐up is 10 months. The exclusion criteria were as follows: (i) open inferior pole of patella fracture; (ii) inferior pole of patella fracture combined with intra‐articular fracture; (iii) patients with previous patellar surgery.

A total of 16 inferior patellar pole fractures were treated at our institution between January 2019 and October 2020, 15 of which underwent treatment with the modified tension band fixation technique consisting of Krachow suturing with Nice knots combined with tension band fixation. One patient treated with Krachow suturing combined with the tension band fixation technique was excluded. Eventually, 15 patients completed at least 1 year of postoperative follow‐up. Among the 15 cases, eight were male and seven were female, with a mean age of 43.4 ± 10.85 years (range: 28–65 years) (Tables 1 and 2).

**TABLE 1:** Clinical and demographic characteristics of patients

Case	Sex	Age(years)	Location	Comminution	Follow‐up time (months)	Bostman score	Knee ROM(°)	Removal of wire
1	M	30	left	Yes	12	30	137.8	Yes
2	F	45	right	No	13	28	129.3	Yes
3	F	53	right	Yes	12	28	122.7	No
4	F	41	left	Yes	14	27	123.8	Yes
5	F	65	left	No	15	27	130.4	No
6	M	28	right	Yes	13	30	143.5	Yes
7	F	46	right	Yes	17	29	122.9	No
8	M	45	right	Yes	12	29	135.5	No
9	M	37	right	No	12	28	145.7	Yes
10	M	58	left	No	16	26	125.0	No
11	F	36	right	No	15	30	134.6	Yes
12	M	57	left	Yes	14	27	127.8	No
13	M	33	left	No	16	28	135.7	Yes
14	M	40	left	No	13	30	152.2	Yes
15	F	37	left	Yes	12	29	113.4	Yes

*Note*: F: female, M: male

**TABLE 2:** Bostman score

Variable	Points	Case(*n*)
Range of movement (ROM)		
Full extension and the ROM >120° or within 10° of the normal side	6	13
Full extension, movement 90°–120°	3	2
Could not be fully extended ROM <90°	0	
Pain		
None or minimal on exertion	6	14
Moderate on exertion	3	1
In daily activity	0	
Work		
Original job	4	15
Different job	2	
Cannot work	0	
Atrophy, difference of circumference of thigh 10 cm proximal to the patella		
<12 mm	4	15
12–25 mm	2	
>25 mm	0	
Assistance in walking		
None	4	12
Cane part of the time	2	3
Cane all the time	0	
Effusion		
None	2	11
Reported to be present	1	4
Present	0	
Giving way		
None	2	10
Sometimes	1	5
In daily life	0	
Stair‐climbing		
Normal	2	15
Disturbing	1	
Disabling	0	
Total score		
Excellent	30–28	11
Good	27–20	4
Unsatisfactory	<20	0

### Surgical Techniques

Anesthesia and position (Step 1): All patients underwent general anesthesia and were placed in the supine position. A pneumatic tourniquet was applied to the thigh to reduce bleeding. The patient received prophylactic intravenous antibiotic therapy (1.5 g of cefuroxime) within 30 min before the tourniquet was inflated.

Approach and exposure (Step 2): A midline incision was made from the superior border of the patella, extending to the tibial tuberosity. The skin flap, consisting of skin and subcutaneous tissue, was lifted during medial and lateral dissection to expose the patellar tendon and fracture. Saline irrigation of the hematoma at the fracture site and in the joint cavity was performed with care to preserve the fracture fragments at the patellar tendon.

**FIG. 1:**
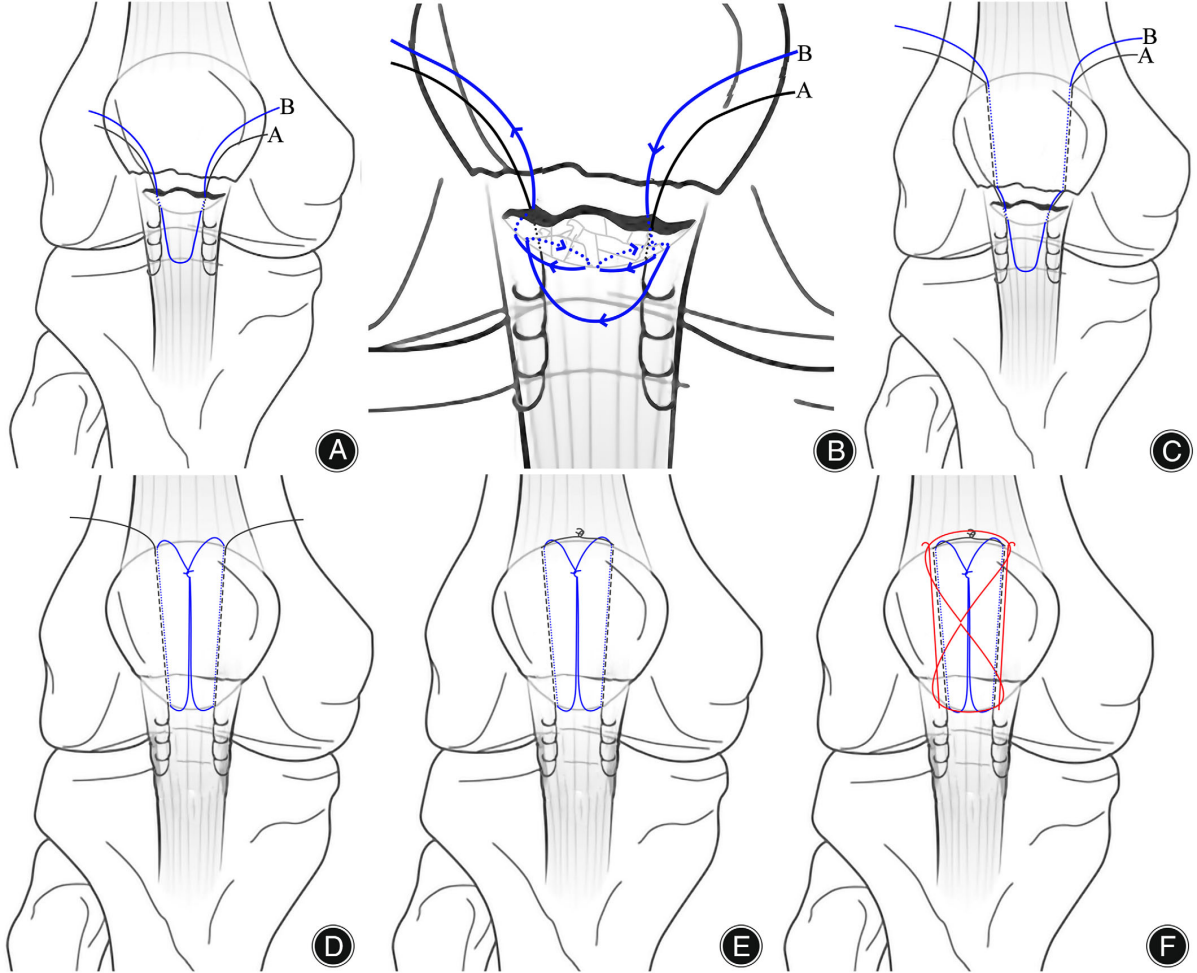
The way line B crosses in a simple fracture is shown in Figure a, while the way line B crosses in a comminuted fracture is shown in Figure b. Line A crosses from both sides of the patellar ligament using Krachow suturing to the patellar bone‐tendon junction, while line B also crosses from this area behind the inferior pole of the patella (A). In cases of comminuted inferior patellar pole fractures, line B is used to form a mesh with line A on the bone‐tendon junction surface using continuous sutures (B). Creation of two bone tunnels in the proximal part of the patella. Lines A and B are introduced through the bone tunnel to the proximal surface of the patella (C). As the inferior pole of the patella is reduced, Line B is tightened using Nice knots to maintain the fracture fragments (D). A surgeon's knot and 3 square knots for tightening Line A (E). Bone pins are implanted under knee flexion to complete the tension fixation technique (F).

**FIG. 2:**
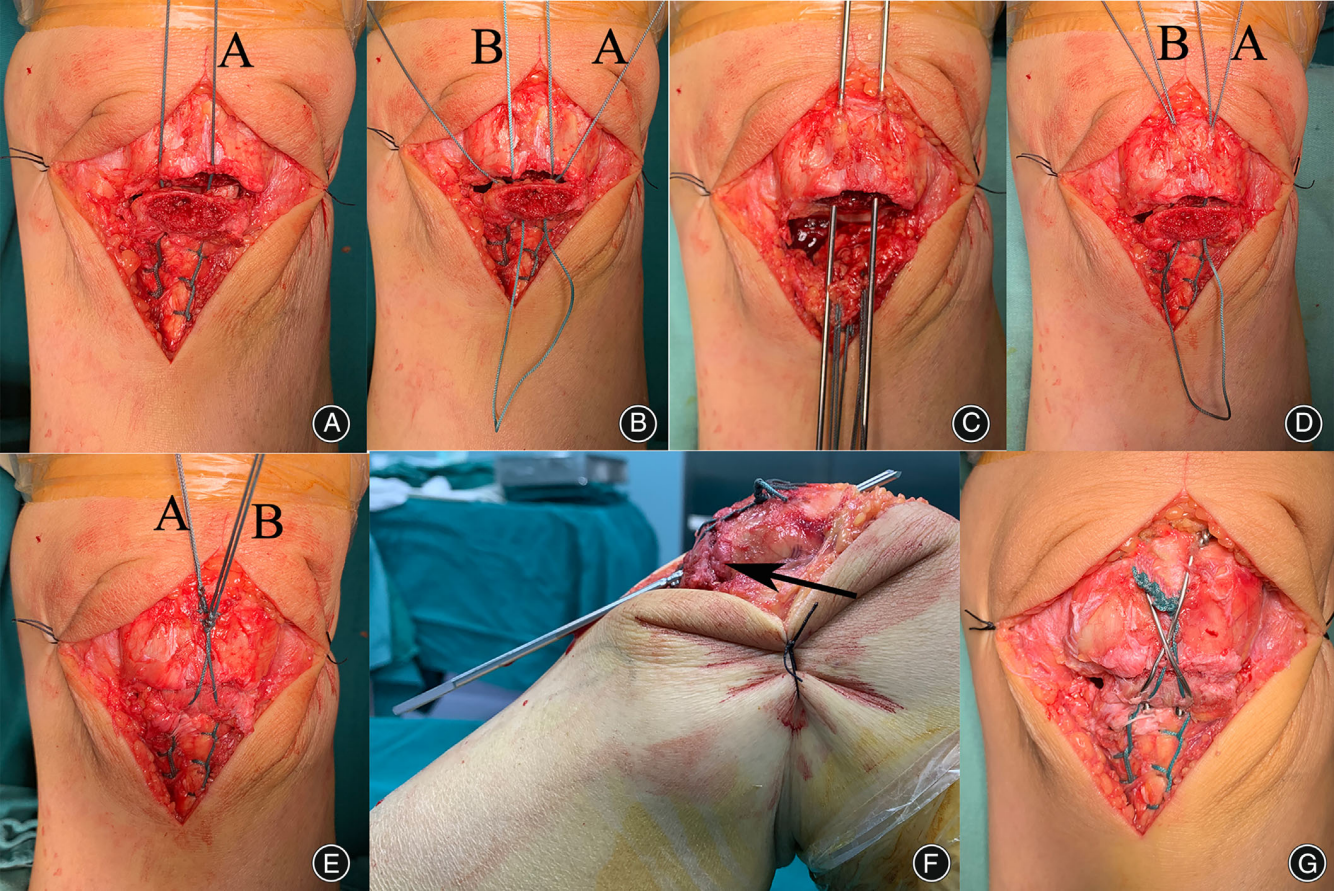
Lines A and B cross behind the inferior pole of the patella from the patellofemoral bone‐tendon junction (A, B).Two bone tunnels are created at the proximal part of the patella with a 2.0 mm Kirschner wire (C). Lines A and B are passed through the bone tunnel (D), and a Nice knot is used to tighten line B after reduction of the fracture fragment (E). A surgeon's knot and 3 square knots are used to tighten line A against the tension of the patellar tendon (E). Bone pins are inserted under knee flexion (F), without displacement of the fracture fragment under flexion (black arrow).

**FIG. 3:**
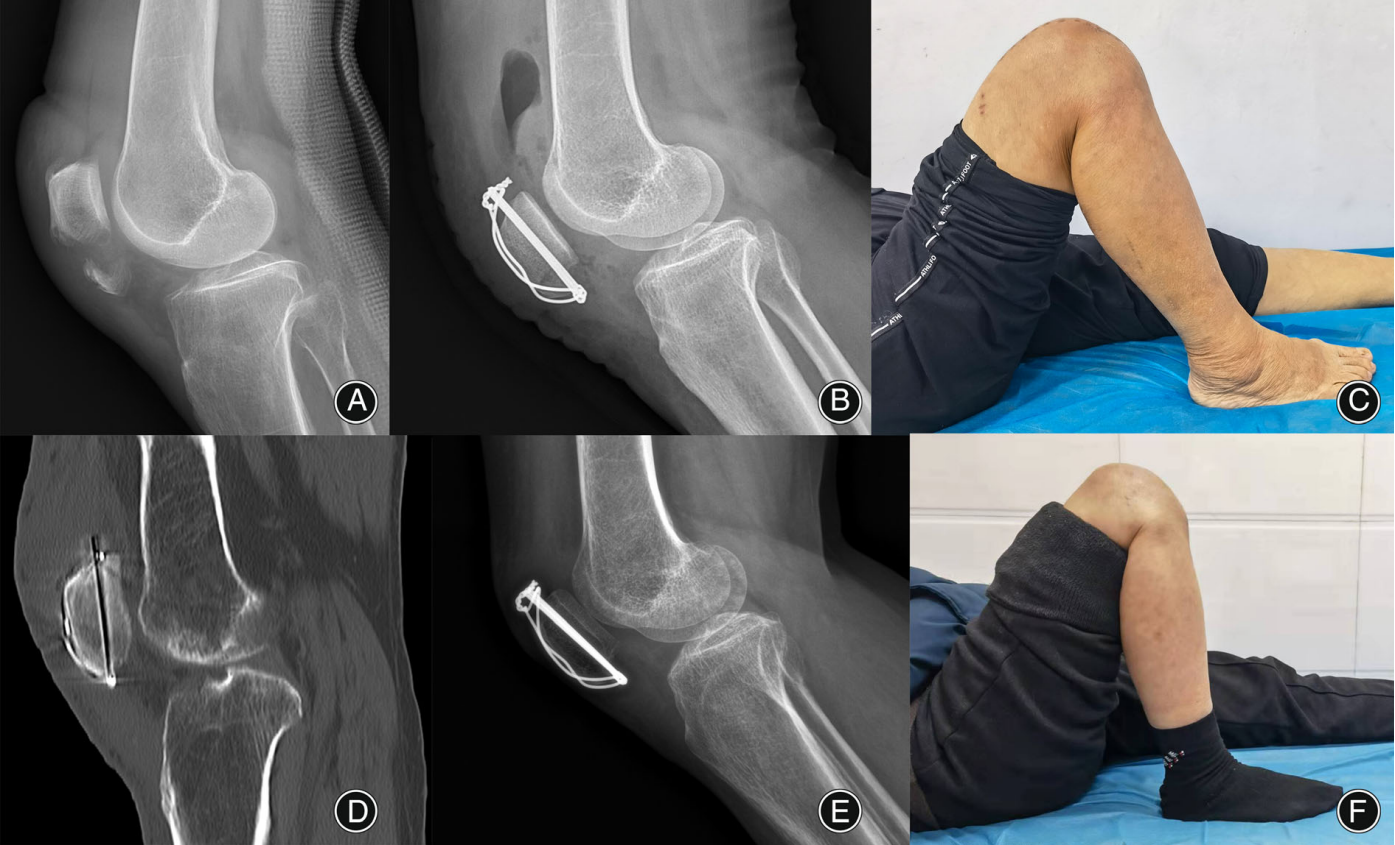
A 53‐year‐old male patient was diagnosed with an inferior patellar pole fracture on radiography (A). Postoperative lateral radiograph of the knee. The bone pin was not inserted through the inferior pole of the patella but below it into the proximal fracture fragment (B). Knee flexion over 90° at 2 weeks postoperatively (C). CT at 9 weeks postoperatively showing complete disappearance of the fracture line with bone union.

Fixation or reconstruction (Step 3): Figure 1 shows the surgical procedure for the modified tension band fixation technique. The first nonabsorbable braided sutures (silk or polyester) were placed along both sides of the patellar ligament using Krachow sutures to the bony surface of the inferior patellar pole and were passed posteriorly from the patellar tendon at the site of bone–tendon union; the second nonabsorbable braided sutures (silk or polyester) were passed directly posteriorly from the site of bone–tendon union of the patellar tendon (Fig. 2A, B). The proximal part of the patella was flipped, and the first bone tunnel was formed by drilling a 2.5‐mm Kirschner wire from the anterior surface of the articular cartilage toward the anterosuperior edge of the patella. In this way, the second bone tunnel was created parallel to the first tunnel, and the width between them was not less than the width of the inferior patellar pole (Fig. 2C). Two sutures (Lines A and B) were passed through the proximal patellar bone tunnel after the lumbar puncture needle was passed (Fig. 2D); the inferior patellar pole was repositioned, and the second suture (Line B) was first fixed and tightened using a Nice knot. Then, the first suture (Line A) was passed through the proximal patella and tied together with a surgeon's knot and three square knots (Fig. 2E). Intraoperatively, the reduction of the fracture was controlled under C‐arm fluoroscopy, and two parallel bone pin holes (2.0 mm) were drilled from the inferior pole of the patella to the superior pole at a distance of 5 mm from the articular surface of the patella at approximately 45–60° of knee flexion (Fig. 2F). A 1.2‐mm wire was then passed through the bone pin hole to create a figure‐eight tension band wiring configuration to apply stress to the fracture (Fig. 2G). Notably, the bone pin was not inserted from the inferior pole of the patella but below it into the proximal fracture fragment (Fig. 3B). Passive knee flexion was performed to determine whether the fracture was stable and whether the internal fixation was secure under direct vision. The joint capsule, subcutaneous tissue, and skin were then closed with absorbable sutures.

### Knee Range of Motion and Bostman Score

The knee range of motion (ROM, degrees) and the Bostman score at 12 months postoperatively were recorded. The Bostman scoring system includes eight sections: range of movement (ROM), pain, work, atrophy difference of the circumference of the thigh 10 cm proximal to the patella, assistance in walking, effusion, giving way, and stair climbing. The Bostman scale is classified into three grades: excellent (30–28 points), good (20–27 points), and unsatisfactory (<20 points)^25^.

## Results

The mean duration of follow‐up was 13.73 ± 1.71 months. When the fracture lines disappeared and the continuity of the trabeculae was confirmed on all X‐rays, bone union was considered complete. Bone union occurred at a mean of 9 weeks postoperatively (range: 8–13). Average hospitalization was 9 ± 1.7 days. There were no cases of postoperative anterior knee pain, refracture of the inferior patellar pole or wire breakage.

### ROM and Bostman Score

The patients regained full ROM of the knee joint without functional deficits during follow‐up; the mean ROM was 128.46° ± 7.07° (range: 113.4°–137.8°). At the latest follow‐up, all patients had a mean Bostman score of 28.40 ± 1.29 (range: 26–30), with an excellent score in 11 patients and a good score in four patients (Table 2).

## Discussion

Fractures of the inferior patellar pole are extraarticular avulsion fractures that do not involve a cartilaginous articular surface, are usually less than 15 mm in vertical length, and do not involve contact with the patellofemoral articular surface during knee flexion or extension^26^. Loss of patellar height due to fracture of the inferior patellar pole disrupts the normal patellofemoral joint anatomy and biomechanical relationships. Therefore, proper treatment of inferior patellar pole fractures is essential to maximize restoration of knee extension and to obtain effective strong internal fixation for early functional exercise and reduce complications such as patellofemoral arthritis^20^, ^21^.

Most scholars believe that the main treatment for inferior pole fractures of the patella is to preserve the length of the patella^1^, ^5^. Matejčić^27^ considered that basket plate treatment of inferior pole fractures of the patella increases the contact area with the bone mass and has better clinical results in his 25 years of clinical experience. With biomechanical studies, KRKOVIC *et al*.^28^ found that the basket plate can cause significant shortening and rupture of the patellar ligament. It is also the provocation of the patellar tendon by internal fixation that has an impact on postoperative functional recovery, such as anterior knee pain. Such plates are not available in many countries. Either technique separate vertical wiring (SVW)^18^, or SVW + patellar wire cerclage^29^ or technique SVW combined with the Krachow suturing^15^ has some clinical effectiveness in the treatment of inferior pole fractures of the patella. However, the wire is unable to convert the tension generated during knee flexion and extension into pressure applied to the fracture site, and in 50% of cases, the bone fragments are displaced forward, followed by deformed healing, causing anterior knee pain and even wire fracture^15^, ^18^, ^29^, ^30^. Meanwhile, 88% of patients have comminuted fractures of the inferior pole of the patella, the comminuted bone mass does not provide sufficient holding force for these surgical approaches, and the internal fixation is easily loosened and falls off^31^.

### Features of the Technique

We propose a modified tension band fixation technique based on the SVW technique. The technique involves converting the tension acting on the anterior surface of the patella into a common pressure on the articular surface. The importance of several technical points cannot be overstated. (1) Krachow suture: The first nonabsorbable braided sutures (silk or polyester) were placed along both sides of the patellar ligament using Krachow sutures to the bony surface of the inferior patellar pole and were passed posteriorly from the patellar tendon at the site of bone–tendon union. (2) The second nonabsorbable braided sutures were passed directly posteriorly from the site of bone–tendon union of the patellar tendon. (3) The surgeon establishes the working channel: The proximal part of the patella was flipped, and the first bone tunnel was formed by drilling a 2.5 mm Kirschner wire from the anterior surface of the articular cartilage toward the anterosuperior edge of the patella. (4) The inferior patellar pole was repositioned, and the second suture (Line B) was first fixed and tightened using a Nice knot. Then, the first suture (Line A) was passed through the proximal patella and tied together with a surgeon's knot and 3 square knots. (5) Bone pin implantation: Two parallel bone pin holes (2.0 mm) were drilled from the inferior pole of the patella to the superior pole at a distance of 5 mm from the articular surface of the patella at approximately 45°–60° of knee flexion.Notably, the bone pin was not inserted from the inferior pole of the patella but below it into the proximal fracture fragment.

**FIG. 4:**
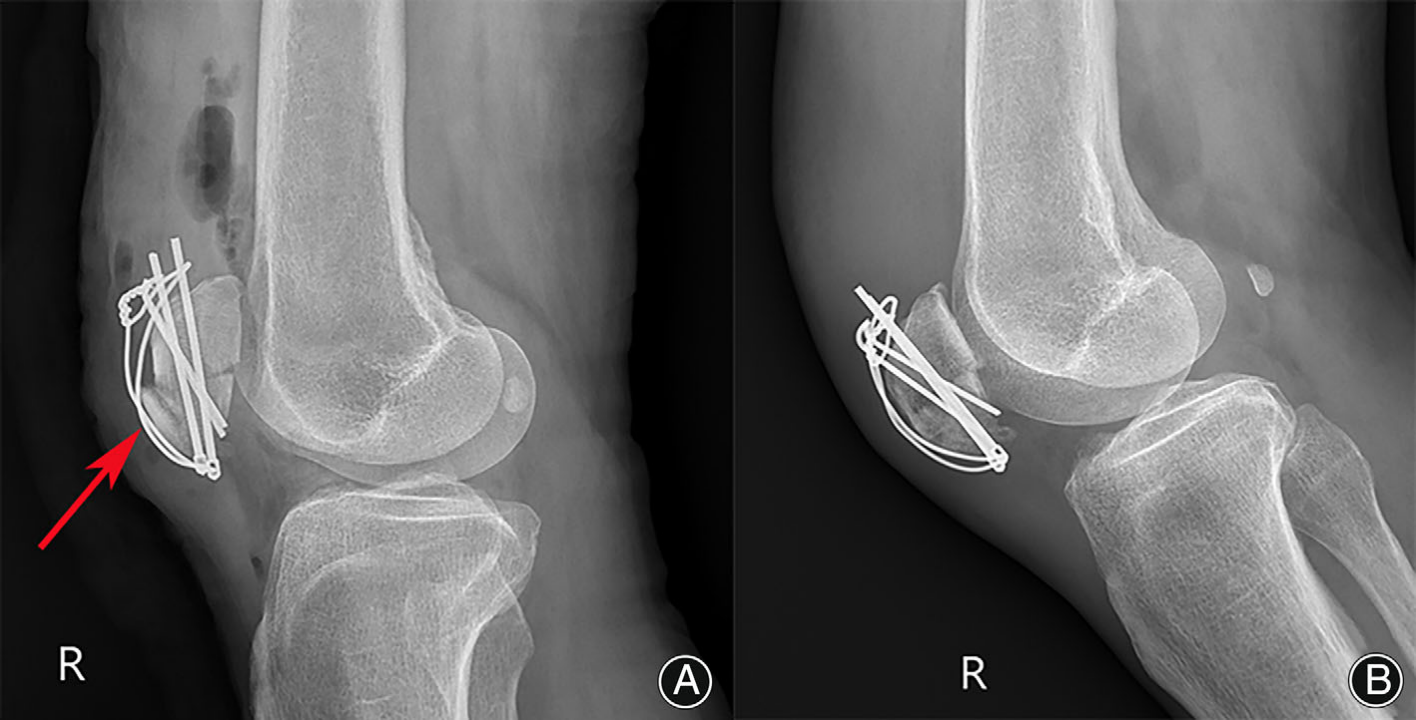
Postoperative lateral radiographs of the knee showing anterior displacement of the fracture fragment at the inferior pole of the patella (red arrow) (A). Lateral radiographs showing blurring of the fracture line and anatomical reduction of the anteriorly displaced fracture fragment at 1 month postoperatively (B).

Biomechanical studies have shown that tension band fixation failure loads of at least 395 N exceed the force of 316 N generated by quadriceps extension.^24^, ^32^ Figure 4A shows the bone pin implanted in the extended position away from the articular surface and at an angle to the articular surface. This is inconsistent with the principle of the tension band fixation technique and is prone to causing avulsion fractures of the patella. Interestingly, postoperative lateral knee radiographs showed anterior displacement of the fracture fragment at the inferior pole of the patella, but at the 1‐month postoperative follow‐up, the anteriorly displaced fracture fragment had regained its anatomical position (Fig. 4). Kim et al.^30^ believe that the SVW technique is unable to translate the tension of the quadriceps into pressure on the patella, which causes anterior displacement of the fracture fragment. Figure 4B shows that anatomical reduction of the fracture fragment was achieved at 1 month postoperatively, and we considered that the tension band fixation technique could explain this phenomenon. Therefore, we modified the SVW technique by using nonabsorbable sutures instead of wire for the Nice knots while adding Krachow sutures for patellar tendon and tension band fixation to improve the stability of fixation and achieve early functional exercise. Professor Boileau^33^ is a famous shoulder surgeon and has described the technique in reconstruction of the rotator cuff. The Nice knot is a novel suture fixation technique proposed by Boileau,^33^ which combines double sutures with slip knots; it has proven useful in the authors' practice over the past 10 years and has replaced wire for fracture fixation. Figure 2B shows two nonabsorbable braided sutures (silk or polyester) inserted through the bone–tendon junction of the patellar ligament and then tightened through the bone tunnel in the patellar body. The Nice knots and Krachow sutures on the coronal plane are distributed appropriately to form a mesh pocket between the sutures so that small fracture fragments do not need to be removed, and in the process of tightening the sutures, even distally crushed bone fragments are repositioned by pulling on the soft tissue without worrying about uneven forces at the tendon‐bone interface of the patellar tendon (Fig. 1A,B). At the same time, the biomechanics provided by the Nice knots allow the implantation of bone pins under knee flexion without fracture displacement (Fig. 2F).^34^ In this position, it is easier to implant the bone pins in close proximity to the patellofemoral articular surface.

### Functional Outcomes

The tension of the Nice knots and tension band fixation achieved with the tightening of the Krachow sutures allow for functional knee flexion and extension without the protection of a knee brace after surgery. In the present study, the modified tension band fixation technique showed good clinical and radiological results. This is a bone‐to‐bone interface reconstruction technique, and the degree and rate of recovery are much greater than those after current tendon‐to‐bone interface reconstruction methods.Patients showed bone healing at an average of 9 weeks postoperatively. At the 1‐year postoperative follow‐up, the knee joint motion was normal, the mean ROM was 128.46° ± 7.07° (range: 113.4°–137.8°), and the Bostman score was excellent, with a mean score of 28.40 ± 1.29. There were no cases of postoperative anterior knee pain, inferior patellar pole refracture or no wire breakage. Among the total number of patients, the four patients over 50 years of age also achieved satisfactory fixation results and adequate fixation strength for early postoperative knee rehabilitation exercises without complications.

### Limitations and Strengths

The Modified tension band technique has the benefits of faster functional recovery of the knee, shorter hospital stay, lower postoperative pain, and lower complication rate. Unfortunately, however, a limitation of this study is that the total number of cases was relatively small, and the method was not compared with other technical methods. There is a lack of biomechanical test results to support these findings. We will need to conduct additional well‐designed studies with large sample sizes to verify the efficacy and safety of the technique.

### Conclusions

We believe that the modified tension band fixation technique (Krachow suturing with Nice knots combined with tension band fixation) for the treatment of inferior patellar pole fractures is a simple and easy‐to‐perform surgical technique that provides stable fixation and good functional results. In future studies, it is possible to use Krachow suturing alone in combination with Nice knots for clinical studies, which could effectively avoid secondary surgery to remove wires or bone pins.

## Author contributions

Cong Xiao designed the study. Zhixiang Gao, Nengji Long, and Kai Yao collected data, writing of the manuscript. All authors were involved in the analysis and interpretation of data, and were involved in writing and revision of the manuscript. All authors approved submission of the manuscript to the journal.

## Conflict of interest

The authors declare that we have no conflict of interest.

## Ethics statement

The authors have no ethical conflict to disclose.
